# Individual Characteristics Associated with Active Travel in Low and High Income Groups in the UK

**DOI:** 10.3390/ijerph181910360

**Published:** 2021-10-01

**Authors:** Emma R. Lawlor, Ruth F. Hunter, Deepti Adlakha, Frank Kee, Mark A. Tully

**Affiliations:** 1 Centre for Diet and Activity Research (CEDAR), MRC Epidemiology Unit, University of Cambridge, Cambridge CB2 0SP, UK; 2 Centre for Public Health, School of Medicine, Dentistry and Biomedical Sciences, Queen’s University Belfast, Belfast BT7 1NN, UK; ruth.hunter@qub.ac.uk (R.F.H.); f.kee@qub.ac.uk (F.K.); m.tully@ulster.ac.uk (M.A.T.); 3 School of Natural and Built Environment, Queen’s University Belfast, Belfast BT7 1NN, UK; deepti_adlakha@ncsu.edu; 4 Department of Landscape Architecture and Environmental Planning, Natural Learning Initiative, College of Design, North Carolina State University, Raleigh, NC 27605, USA; 5 Institute of Mental Health Sciences, School of Health Sciences, Ulster University, Newtownabbey BT37 0QB, UK

**Keywords:** active travel, income, walking, cycling

## Abstract

Active travel (AT) has gained increasing attention as a way of addressing low levels of physical activity. However, little is known regarding the relationship between income and AT. The aim of this study was to investigate characteristics associated with undertaking AT in an adult population and by low- and high-income groups. Data collected from the Physical Activity and the Rejuvenation of Connswater (PARC) study in 2017 were used. Participants were categorised into socio-economic groups according to their weekly household income, and were categorised as participating in ‘no’ AT or ‘some’ AT and ‘sufficient’ AT. Multivariable logistic regression explored characteristics associated with AT in the full cohort, and the low- and high-income groups separately. Variables associated with AT in the low-income group were body mass index (BMI), physical activity self-efficacy, marital status, long term illness, difficulty walking and housing tenure. For the high-income group, BMI, marital status, housing tenure and education were associated with AT. For both income groups, there were consistent positive associations with the action/maintenance phase of the stage of change model across all AT categories. The findings suggest that population sub-groups may benefit from targeted initiatives to support engagement in AT and prevent further widening of inequalities.

## 1. Introduction

Insufficient physical activity (PA) is the fourth leading cause of mortality globally [1], and is associated with large direct and indirect economic consequences for society [2,3,4,5]. Despite the strong evidence for the health benefits of engaging in PA, such as decreasing risk of non-communicable diseases and all-cause mortality [1,6,7,8,9], it is estimated that 45% of adults (≥16 years) in Northern Ireland [10] do not achieve the current recommended level of 150 min of PA per week [11]. Furthermore, Hallal and colleagues [12] estimated that physical inactivity levels (≥15 years) in the United Kingdom (UK) were 63.3%, considerably higher than the European average of 34.8%. Understanding more about the different domains of PA (e.g., active travel (AT), leisure, occupational) can have important implications for policy, clinical practice and future public health investments [13,14].

AT is walking or cycling as an alternative to motorised transport for the purpose of making everyday journeys [15]. AT is an accessible method of incorporating PA into daily life [16,17]. It has been shown to improve health [8,18,19,20,21,22], with individuals who engage in regular AT being at a lower risk of all-cause mortality and cardiovascular disease and diabetes incidence [22]. Engagement in AT also has wider societal benefits, such as a reduction in transport costs, costs to healthcare services, pollution and traffic congestion [23,24]. Moreover, adults who engage in AT travel are more likely to meet PA guidelines than those that use motorised modes of transport [16,25,26,27]. However, in many countries, levels of AT are very low [28,29,30,31]. Currently, approximately 42% of all journeys in the UK are under two miles [32]; this is an ideal opportunity to promote engagement in AT either for the whole or part of the journey in combination with other transport modes.

Socio-economic position (SEP) is a known factor to influence health and engagement in health promoting behaviours [33,34]. Due to it being easily accessible and having a low cost, AT could potentially reduce inequalities [35], but there are inconsistent findings regarding the influence of SEP on engagement in AT. For example, a systematic review by Beenacker et al. [36] found an inconsistent pattern between SEP and AT, whereas Adams [37] found a negative association between being sufficiently active through AT and lower SEP. Furthermore, Olsen et al. [38] found that people living in the most deprived areas in Scotland were more likely to report AT than those in the least deprived areas. In these previous studies, SEP has been assessed using information including income (individual or household), occupation-based social class and area-based deprivation measures [36,37,38]. Despite this gap in knowledge, previous studies on AT have tended to include populations living in an area of a single SEP, or it was beyond the realm of the study to investigate factors related to SEP.

Socio-demographic, health, environmental and psychological factors have been found to potentially influence AT behaviour. However, most research on these determinants of AT has also focused exclusively on commuting to work or school, and excluded those not in employment [16,17,18,27,39,40,41]. Approximately 30.7% of the Northern Ireland adult population (≥16–64 years) are not in employment [42], and 24.8% in the UK (≥16–64 years) are economically inactive [43]. As AT encompasses non-work-related journeys (e.g., shopping, errands) [15], AT can be undertaken by those not employed therefore previous studies have disregarded a sizable proportion of the population. It is also important to investigate AT specifically, as factors influencing AT may differ compared to other types of PA (e.g., leisure, occupational, commuter). AT has previously been assessed in studies most frequently using self-reported measures, such as study-specific questionnaires, validated PA questionnaires, travel diaries and nationally representative surveys or census data [13,17,18,36,37,38,39,40,41,44]. 

Policy makers recognise that the effects of environmental AT interventions vary between different populations and SEP, and thus this needs to be further understood [44,45]. Recently, AT has gained heightened recognition from policy makers within the UK [46]. For example, Northern Ireland has established AT targets, aiming for 20% of all journeys less than 1 mile to be cycled by 2025, and 40% by 2040 [47]. The UK’s government has also set out aims in their ‘Cycling and Walking Investment Strategy’ to increase the number of stages walked and doubling cycling levels (to 1.6 billion stages) by 2025 [48]. These ambitious aims strengthen the need for further exploration to identify key sub-populations that may need targeted AT initiatives and additional support.

This study will help to identify these sub-population groups and contribute evidence to increase understanding of the relationship between SEP and AT. It will also provide insights into the influence of socio-demographic, health, environmental and psychological factors with AT in differing income groups. Understanding of these factors and relationships between SEP and AT can help to inform AT interventions that do not further widen inequalities and will potentially increase levels of AT and PA. Thus, the aim of this study was to investigate characteristics associated with undertaking AT in an adult population and by low and high income.

## 2. Materials and Methods

### 2.1. Survey

This paper used data from the Physical Activity and the Rejuvenation of Connswater (PARC) study [49], a natural experiment investigating the public health impact of the creation of the Connswater Community Greenway (CCG). The target area of the survey was the 29 electoral wards (population of approximately 110,600) within a one-mile radius of the greenway. This area consists of a wide range of socio-economic positions, with 7 of these wards being within the top 25% most deprived in Northern Ireland [50].

The survey was completed over a 12-month period after the development of the Greenway in 2017. The survey was interviewer administered with the sampling strategy based on evidence of the number of residents in each ward aged 16 or over provided by a national census in 2011. A random probability sampling framework was utilised to choose a random selection of addresses from the Royal Mail Postal Address File, stratified by the proportion of the overall population within each area. The ‘last birthday rule’ (the individual aged 16 or over who most recently had their birthday) was used to select an individual from within the household to complete the survey. An information sheet about the study was posted to each household then followed up 1 to 2 weeks later by a trained interviewer to gain written consent and administer the questionnaire. All questions were self-reported by the interviewee.

### 2.2. Outcome Measures

#### 2.2.1. Dependent Variable

Active travel: Participants were asked about their frequency and duration of PA in a typical week using the Global Physical Activity Questionnaire (GPAQ) [51,52], creating a continuous variable of time spent in AT (minutes/week). Using these data, individuals were also categorised into the groups: those participating in no AT (0 minutes/week); those who participated in ‘some AT’ (≥10 minutes/week); those sufficiently active through AT (≥150 minutes/week), i.e., meeting PA recommendations.

#### 2.2.2. Independent Variables

Independent variables were grouped into socio-demographic, health, environmental and psychological factors. Independent variables were included based on existing literature suggesting that they may be associated with AT.

Socio-demographic characteristics: Data were collected for gender, age, marital status, housing tenure, household income per week, education, access to bicycle (‘yes’ or ‘no’), and car ownership. Household income per week was categorised in tertiles: low (£60–£230 per week), medium (£231–£580/week) and high (£581+ per week). Highest education achieved was grouped into ‘Primary—none/other qualifications’, ‘Secondary—GCSE/A-level’ and ‘Tertiary—Degree/higher education’. Employment status was either ‘Employed’ or ‘Unemployed/economically inactive’. All variables were categorical apart from age which was continuous, and access to bicycle was binary (yes/no).

Health variables: Data were collected for BMI, long term illness which limits PA (‘yes’ or ‘no’) and difficulty walking a quarter mile (‘yes’ or ‘no’). Self-reported height and weight was used to calculate BMI (weight (kg)/height (m)^2^) that was then categorised into underweight/normal (<24.9 kg/m^2^) and overweight/obese (>25 kg/m^2^). Health assessments included the Short Form-8 Health Survey (SF-8) [53], generating a physical and mental component score and the Warwick–Edinburgh Mental Wellbeing Scale (WEMWBS) [54], with higher scores indicating better self-rated physical and mental health, and mental well-being, respectively.

Environmental factors: The data generated for this variable were the distance travelled to work daily. These data were categorised into ‘Do not work or study/do not work’ and ‘Travel any distance to work’.

Psychological factors: Psychological variables were the Physical Activity Self-Efficacy mean score [55] and the Physical Activity Stages of Change questionnaire [56], with the three possible categories of ‘Pre-contemplation’, ‘Contemplation/preparation’ and ‘Action/Maintenance’. These were categorised due to low numbers in the contemplation, preparation and action stages.

#### 2.2.3. Statistical Analysis

All statistical analyses were completed using STATA version 14. Descriptive statistics for the full cohort, and low- and high income-groups were calculated. For categorical variables, the descriptive statistics were the number (*n*) and percentage (%) of participants in each category. For continuous variables, the descriptive statistics used were mean and standard deviation (SD).

The association between variables and each of the three AT participation groups was explored using separate regression models. In these analyses, all variables were entered simultaneously. Age, WEMWBS, SF8 Mental Score, SF8 Physical Score and Physical Activity Self-Efficacy were entered as continuous variables, with all other variables being binary or categorical. Multiple logistic regression was used for the binary AT variable (any AT) and a separate multiple logistic regression was conducted for the binary variable of being sufficiently active through AT (≥150 minutes/week). Multiple linear regression was used for the continuous AT variable (time spent on AT, if any). Only participants with complete data were included in the models. This was completed for the full cohort, and low- and high-income groups separately to enable comparison between the two income groups.

A sensitivity analysis to test for missing data using multiple imputation chain equations was conducted for the full cohort. Data were imputed for the variables BMI, income, PA self-efficacy and marital status. This sensitivity analysis was not conducted for the low- and high-income groups as the amount of data missing was negligible.

## 3. Results

### 3.1. Participant Characteristics

The full cohort consisted of 1214 participants, with 916 participants having complete data. For the full cohort, participants had a mean age of 51.7 years (SD 19.1), and just over half of the sample were women, had overweight or obesity and were in employment. Further, 30.6% had access to a bicycle, 72.0% had access to a car and 31.6% had a long-term illness limiting activities. In this cohort, 64.1% (*n* = 778) reported doing ‘some AT’ in the past week and had a median of 90 minutes of AT per week.

The full cohort was divided by income, with 25.1% (*n* = 244) in the low-income group and 29.5% (*n* = 286) in the high-income group. Over half the individuals in both the low- and high-income groups were women but, some differences in characteristics were evident between groups. In comparison to the high-income group, the low-income group had an older sample, a higher number of single/separated/divorced/widowed individuals, home renters, individuals experiencing a long term illness that limited activities and had difficulty walking a quarter mile. The high-income group had a higher proportion of people needing to travel a distance to work, owned at least one car, had access to a bicycle, had tertiary education and were in employment than the low-income group. Further, the high-income group had higher mean scores for psychological outcomes and the majority were in the action/maintenance phase of the stages of change model in contrast to the low-income group (Table 1). For AT, a larger number in the high-income group reported doing ‘some AT’ in the past week, in comparison to the low-income group (72.7; *n* = 208 vs. 53.7; *n* = 131, respectfully) (Table 2). The findings for medium income are in Tables S1–S5.

### 3.2. Associations with Active Travel

#### 3.2.1. Engaging in Some Active Travel

The multiple logistic regression model exploring the association between socio-demographic variables and some AT (≥10 minutes per week) is summarised in Table 3. For the full cohort, there was a decreased likelihood of engaging in some AT for those who were of underweight/normal weight compared to those with overweight/obesity, having difficulty walking a quarter of a mile, and being in the pre-contemplation or contemplation/preparation phases of the stages of change model compared to being in the action/maintenance phase. Not owning a car was associated with increased likelihood of some AT compared to owning two or more cars. WEMWBS score was also positively associated with increased likelihood of some AT. The pseudo-R^2^ value suggests that individual factors explained 15.0% of the variance in AT.

Differences were found by income group. For the lower income group, being under/normal weight compared to having overweight/obesity and being in the pre-contemplation or contemplation/preparation phase of the stages of change model rather than the action/maintenance phase were associated with decreased likelihood of AT, with variance of 32.0%. For the high-income group, being in the contemplation/preparation phase compared to the action/maintenance phase was also associated with being less likely to take part in AT and had a variance of 10.0%.

#### 3.2.2. Amount of Time Engaging in Active Travel

Table 4 summarises the multiple linear regression analysis exploring the relationship between the socio-demographic variables and time spent on AT. For the full cohort, being in the pre-contemplation or contemplation/preparation phases of the stage of change model were significantly associated with less time spent in AT compared to the action/maintenance phase, and SF8 physical summary score was positively associated with AT time. The R^2^ value indicated that individual factors explained 11.0% of the variance in AT.

For the low-income group, being in the contemplation/preparation phase was associated with less time in AT than the maintenance/action phase, but PA self-efficacy was positively associated with time in AT, with 28.0% variance. For the high-income group, being married/co-habiting increased time spent in AT compared to being single/separated/divorced/widowed, while owning a house outright or having a mortgage/co-owning a house was associated with less time spent in AT than those who rented/other, with a variance of 18.0%.

#### 3.2.3. Sufficiently Active through Active Travel

Table 5 summarises the multiple logistic regression analysis exploring the relationship between the socio-demographic variables and being sufficiently active through AT (>150 minutes per week). For the full cohort, age was negatively associated with being sufficiently active through AT. Those less likely to be sufficiently active through AT were those with difficulty walking a quarter mile compared to those without any difficulty, and those in the pre-contemplation or contemplation/preparation phases of the stage of change model compared to being in the action/maintenance phase. Not owning a car was associated with increased likelihood of being sufficiently active through AT compared to owning more than one car, and SF8-physical summary score was positively associated with being sufficiently active. The pseudo-R^2^ value suggests that individual factors explained 16.0% of the variance in AT. For lower income group, individuals with a mortgage/co-owning a house compared to renting/other, those married/co-habiting rather than being single/separated/divorced/widowed and those reporting a long-term illness limiting activities were more likely to be sufficiently active through AT. However, those with difficulty walking, and individuals in the pre-completion or contemplation/preparation phases of the stages of change model rather than in the action/maintenance phase were less likely. Variance was 34.0%. For high income, being under/normal weight was associated with increased likelihood of being sufficiently active through AT compared to those with overweight/obesity. Those with a decreased likelihood of being sufficiently active through AT were those with secondary education compared to those with tertiary education, and those in the contemplation/preparation phase rather than the action/maintenance phase of the stages of change model. The variance was 11.0%.

## 4. Discussion

This study found specific characteristics associated with AT, which differed between low- and high-income groups. The key variables found to be associated with engaging in AT in the low-income group were BMI, marital status, housing tenure, having a long-term illness limiting activities, difficulty walking and PA self-efficacy. For the high-income group, the results suggest that BMI, marital status, housing tenure and education were significantly associated with engaging in AT. For both income groups, there were consistent positive associations with being in the action/maintenance phase of the stage of change model across all AT categories.

Our study found a large number of people engaging in some AT (64%) (and being sufficiently active through AT (40%))—higher than the findings of other studies [37,38,40,44]. This may be explained by including non-work commuting which has been previously overlooked in studies, or as we used a self-report questionnaire which can have validity and reliability issues [57]. The low-income group had a lower median minutes of AT per week; these respondents were older, included more females, and reported higher levels of long-term illness and difficulty walking than the high-income group, all of which are known to decrease engagement in PA [12,58]. Indeed, for the whole cohort, our findings reflect previous evidence that age is negatively associated with AT and PA [13,18,37,38,39,40,44].

Despite the intuitive connection and in contrast to previous research [44], no associations between AT and owning a bicycle was evident. However, similar to Northern Ireland levels [59], almost a third of those surveyed owned a bicycle. This suggests that cycling may be an acceptable form of AT and many already have the necessary equipment. However, they may also be only used for leisure and barriers to their use for AT may exist [60]. Previous studies [61,62] in a similar location in Belfast found that barriers to PA (including AT) included vandalism, dogs, apathy, poor weather, lack of facilities, urban infrastructure and paramilitary activity. A range of policies and initiates could overcome these barriers and encourage using the urban greenway for AT.

Ogilvie et al. [44] previously found in a deprived population that living in owner occupied accommodation was associated with AT. Our findings suggest for the low-income group that having a mortgage or co-owning a house increased the likelihood of being sufficiently active; individuals in this income group may have engaged in AT as it is cost-free, enabling income to be spent on housing instead. In contrast, for the high-income group, owning outright, and co-owning or having a mortgage in comparison to renters decreased the time likely spent in AT, potentially due to more individuals in this income group having access to a car. Less evident in previous research, our findings found marital status to be influential, as well as becoming significantly associated with being sufficiently active when data were imputed. This suggests that housing tenure and marital status are important, but often overlooked correlates of AT and warrant further investigation.

Leaving full time education at an older age has previously been positively associated with AT [37]; for the high-income group, our study found similar findings with those with a higher level of education being more likely to be sufficiently active through AT. This association with education is common for multiple health outcomes and behaviours [63], although our findings suggest that it is most beneficial for AT interventions to target those with low educational attainment but higher income.

Unlike previous evidence [17,37,39,44], our study did not find any associations between employment related variables and AT; Adams [37] found using the UK time use survey that those employed were less likely to be sufficiently active, plus multiple studies [17,39,44] suggested that living closer to work is associated with AT, although comparisons are limited as they excluded those unemployed and only investigated commuting behaviour. Other employment-related factors beyond the scope of this study may be important, such as needing a car for work, travelling to other places before/after work and parking difficulties/cost of parking at work [39,44], and other neighbourhood environmental factors [29]. Furthermore, there is a need for future AT research to consider those unemployed and how they can be supported.

Consistent with previous findings [17,37,39,44], for the full study cohort, not owning or having access to a car was found to be a significant predictor of AT. This may be out of necessity or due to increased use of public transport as users tend to engage in more walking [64,65]. The findings may suggest that interventions that promote AT as an alternative to the car, or make car ownership more difficult (e.g., reduced parking, taxes), could be successful. However, this was not evident by income group; potentially the analyses may have been underpowered to detect an association.

Income was particularly important when unpicking associations with BMI, with contrasting findings between income groups. It is also not possible to define if associations are cause or effect. Previous evidence has found that those with overweight or obesity was associated with not engaging in AT [18]. Mytton et al. [66] also found that those who regularly cycled to work or incorporated AT into longer journeys had lower adipose levels; as this included only work commuters, this may reflect our finding for the high income group of being sufficiently active as more people were in employment. Further investigation is potentially needed for the potential of recommending AT for weight management.

For the low-income group, echoing previous evidence [44] and rather intuitively, difficulty walking was negatively associated with being sufficiently active. However, our finding that having a long-term illness increased likelihood of engaging in AT may suggest that it could be an acceptable way to encourage those with chronic conditions (particularly those without difficulty walking) and a lower income to increase their PA. The SF8 physical summary score being positively associated with AT for the full cohort aligns with Bopp et al.’s [18] finding that better perceived health was associated with walking and cycling. The individual’s perception of their health potentially should therefore be taken into account when designing interventions, although this was not evident by income group or for perceived mental health.

Self-efficacy for PA was associated with engaging in AT for the lower income group, suggesting that interventions that target individuals with high self-efficacy for PA in low SEP groups are worth exploring. WEMWEBS was only found to be associated with increased likelihood of doing AT in the full cohort.

Being in the action/maintenance phase of the stages of change model was most consistently associated with better levels of AT. A slight discrepancy was that there was no association with increased minutes of AT for the high-income group. This may be due to engaging in other forms of PA rather than AT, but previous research [67] has found that engagement in AT was not negatively associated with recreational PA. Our findings suggest that interventions to assist people in moving into the action/maintenance phase of the model could be essential to improving levels of AT.

These results have important public health research, practice and policy implications [13,14]. The differences in results between the full cohort and different income groups highlight that when designing interventions there is a need to appreciate that ‘one size does not fit all’, and the individual and local context should be taken into consideration. Further, individuals that already engage in ‘some’ AT could be a key group in improving population levels of PA through AT and should receive targeted support.

### Strengths and Limitations

This study provides new insights into the association of high and low income with AT. This study included respondents currently unemployed or economically inactive which has been overlooked in other studies, reflecting the population of the area and considering non-commuting AT. A limitation of this study was that BMI was calculated from subjective self-reported height and weight data, and PA was also based on self-report, as no objective assessment was available. The respondents were mostly women, thus future studies should attempt to include more men in their sample. By dividing the sample into the different SEP groups, this reduced the power of the sample therefore future studies with larger sample sizes is warranted. A large number of participants reported they engaged in no minutes of AT; this may be due to recall bias therefore an objective PA measurement could improve reliability. Furthermore, some of these participants may have engaged in less than 10 min bouts, but this was not captured using this questionnaire as at the time of data collection, the recommendations were for PA to be in bouts of 10 minutes. Furthermore, it needs to be acknowledged that there may be residual confounding from variables that we did not include in our models, and that there may be some instances of reverse causality. Future studies could investigate the impact of different variables on AT by income that were beyond the realm of our study.

## 5. Conclusions

This study found that a variety of individual and household, psychological and environmental factors were associated with AT, with differences between income groups present. Key variables appeared to be BMI, difficulty walking, having a long-term illness, PA self-efficacy, marital status, housing tenure, education and phase of stages of change model. This suggests that many complex, interacting factors influence engagement in AT that need to be addressed in future initiatives. Our results provide some practical implications, such as that interventions to promote AT may be most beneficial to those with high self-efficacy in low SEP groups and those of high income with low educational attainment, and for individuals of all income levels to be supported to move into the action/maintenance phase of the stage of change model. The findings also highlight the need for more investigation in relation to income into how housing and marital status impact AT, and AT’s relationship with weight management and other factors beyond the realm of our study that may influence AT (e.g., work-related and environmental). Further investigation is required to enable the construction of effective interventions targeting sub-groups of the population, and there is a need for greater understanding of reasons influencing the choice to engage in AT by income. These findings can inform the development of future AT interventions and have important implications for public health policy and practice to address inequalities in AT participation.

## Figures and Tables

**Table 1:** Participant socio-demographics for full cohort and by low and high income.

Variable	Full Cohort (*n*= 1214)		Low Income (*n*= 244)		High Income (*n*= 286)	
	N	%	N	%	N	%
Sex						
Male	531	43.74	85	34.84	132	46.15
Female	683	56.26	159	65.16	154	53.85
Age						
16 to 39 years	393	32.37	60	24.59	101	35.31
40 to 64 years	471	38.80	74	30.33	156	54.55
65+ years	350	28.83	110	45.08	29	10.14
Mean (years (SD)):	51.66 (19.07)	-	57.66 (20.30)	-	46.02 (13.91)	-
Range (years):	16–99	-	19–96	-	18–88	-
Marital status *						
Married/co-habiting	571	47.19	45	18.44	218	76.22
Single/Separated/divorced/widowed	639	52.81	199	81.56	68	23.78
Housing tenure						
Owned outright	405	33.36	67	27.46	87	30.42
Mortgage/co-ownership	333	27.43	13	5.33	155	54.20
Rented/other	476	39.21	164	67.21	44	15.38
Weekly household income *						
Low Income (£60–£230)	244	25.13	-	-	-	-
Medium Income (£231–£580)	441	45.42	-	-	-	-
High Income (£581+)	286	29.45	-	-	-	-
BMI category *						
<24.9 Underweight/Normal	539	46.07	111	47.44	122	43.88
25–29.9 Overweight/> 30 Obese	631	53.93	123	52.56	156	56.12
Highest education						
Primary—none/other qualifications	226	18.62	99	40.57	7	2.45
Secondary–GCSE/A-level	472	38.88	118	48.36	69	24.13
Tertiary—degree/higher education	516	42.50	27	11.07	210	73.43
Access to bicycle						
Yes	371	30.56	32	13.11	162	56.64
No	843	69.44	212	86.89	124	43.36
Number of cars/vans owned						
0	340	28.01	151	61.89	11	3.85
1	543	44.73	84	34.43	111	38.81
2+	331	27.27	9	3.69	164	57.34
Employment						
Employed	608	50.08	38	15.57	233	81.53
Unemployed/economically inactive	606	49.92	206	84.43	53	18.47
Long term illness limiting activities						
Yes	383	31.55	148	60.66	44	15.38
No	831	68.45	96	39.34	242	84.62
Difficulty walking a quarter mile						
Yes	268	22.08	108	44.26	26	9.09
No	946	77.92	136	55.74	260	90.91
Distance travelled to work daily						
Do not work or study/Work at home	599	49.34	206	84.43	61	21.33
Travel any distance to work	615	50.66	38	15.57	225	78.67
Active travel						
None	436	35.91	113	46.31	78	27.27
Some	778	64.09	131	53.69	208	72.73
Stage of change						
Pre-contemplation	394	32.45	125	51.23	49	17.13
Contemplation/Preparation	187	15.40	29	11.89	59	20.63
Action/Maintenance	633	52.14	90	36.89	178	62.24
	Mean	SD	Mean	SD	Mean	SD
WEMWBS	51.21	9.30	45.58	10.05	53.98	7.00
SF8 Mental Summary Score	48.75	10.24	43.94	11.65	50.88	7.35
SF8 Physical Summary Score	48.18	10.84	42.49	11.37	52.14	7.83
Physical Activity Self—Efficacy Mean *	2.38	0.97	1.93	0.93	2.71	0.96

* Participant data missing, therefore the total does not equal 1214. (N for variables with missing data for full cohort: marital status: *n* = 1210; socio-economic position: *n* = 971; BMI category: *n*= 1170; physical activity self-efficacy mean: *n*= 1176).

**Table 2:** Engagement in active travel by participants for full cohort and by low and high income.

	Full Cohort	Low Income	High Income
Median minutes of active travel per week (minutes (IQR))	90 (0–240)	30 (0–205)	120 (0–240)
Number of participants doing ‘some’ active travel (over 10 minutes per week) (*n* (%))	778 (64.09)	131 (53.69)	208 (72.73)
Median minutes of active travel for those that do ‘some’ active travel (over 10 minutes per week) (min (IQR))	200 (100–315)	160 (90–360)	200 (100–300)
Number of participants that achieve over 150 minutes of active travel per week (*n* (%))	489 (40.28)	75 (30.74)	125 (43.71)

IQR: Inter-quartile range.

**Table 3:** Multiple logistic regression of socio-demographic, health, environmental and psychological associations of none/some active travel (binary) for full cohort and by low and high income.

Variable	Full Cohort (*n* = 916) *	Low Income (*n* = 219) *	High Income (*n* = 277) *
	Odds Ratio (95% CI)	Odds Ratio (95% CI)	Odds Ratio (95% CI)
Sex			
Male	0.98 (0.71, 1.36)	1.99 (0.89, 4.45)	1.09 (0.59, 2.01)
Female	Ref.	Ref.	Ref.
Age (continuous, per year)			
	0.98 (0.97, 1.00)	0.98 (0.96, 1.01)	1.00 (0.97, 1.03)
Marital status			
Married/co-habiting	1.07 (0.73, 1.57)	2.95 (0.99, 8.80)	0.46 (0.19, 1.09)
Single/Separated/divorced/widowed	Ref.	Ref.	Ref.
Housing tenure			
Owned outright	1.40 (0.86, 2.28)	1.30 (0.51, 3.34)	1.33 (0.38, 4.61)
Mortgage/co-ownership	1.21 (0.75, 1.97)	1.43 (0.24, 8.46)	1.08 (0.40, 2.96)
Rented/other	Ref.	Ref.	Ref.
Weekly household income			
Low Income (£60–£230)	1.39 (0.76, 2.54)	-	-
Medium Income (£231–£580)	1.00 (0.65, 1.53)	-	-
High Income (£581+)	Ref.	-	-
BMI category			
Underweight/Normal	0.65 (0.48, 0.90)	0.38 (0.17, 0.82)	1.35 (0.73, 2.52)
Overweight/Obese	Ref.	Ref.	Ref.
Highest education			
Primary—none/other qualifications	0.65 (0.39, 1.09)	1.31 (0.35, 4.95)	1.29 (0.13, 13.27)
Secondary—GCSE/A-level	0.84 (0.58, 1.21)	1.43 (0.40, 5.08)	0.72 (0.37, 1.38)
Tertiary—degree/higher education	Ref.	Ref.	Ref.
Access to bicycle			
Yes	1.25 (0.84, 1.84)	0.40 (0.10, 1.58)	1.70 (0.93, 3.13)
No	Ref.	Ref.	Ref.
Number of cars/vans owned			
0	1.91 (1.04, 3.50)	3.41 (0.43, 26.71)	5.20 (0.45, 60.33)
1	1.34 (0.87, 2.06)	2.13 (0.31, 14.77)	1.49 (0.75, 2.98)
2+	Ref.	Ref.	Ref.
Employment			
Unemployed/economically inactive	1.04 (0.41, 2.60)	0.22 (0.02, 2.57)	1.26 (0.29, 5.54)
Employed	Ref.	Ref.	Ref.
Long term illness limiting activities			
Yes	0.99 (0.59, 1.65)	1.54 (0.49, 4.86)	0.69 (0.25, 1.92)
No	Ref.	Ref.	Ref.
Difficulty walking a quarter mile			
Yes	0.56 (0.33, 0.94)	0.62 (0.24, 1.58)	1.39 (0.38, 5.04)
No	Ref.	Ref.	Ref.
Distance travelled to work daily			
Do not work or study/Work at home	1.12 (0.45, 2.76)	3.00 (0.28, 32.18)	0.98 (0.24, 3.93)
Travel any distance to work	Ref.	Ref.	Ref.
Stage of change			
Pre-contemplation	0.49 (0.32, 0.74)	0.33 (0.12, 0.92)	0.68 (0.30, 1.52)
Contemplation/Preparation	0.45 (0.29, 0.70)	0.27 (0.08, 0.89)	0.48 (0.23, 0.98)
Action/Maintenance	Ref.	Ref.	Ref.
WEMWBS (continuous, per unit)	1.04 (1.01, 1.06)	1.05 (1.00, 1.11)	1.05 (0.99, 1.10)
SF8 Mental Summary Score (continuous, per unit)	1.01 (0.99, 1.03)	1.01 (0.96, 1.06)	1.02 (0.98, 1.07)
SF8 Physical Summary Score (continuous, per unit)	1.01 (0.99, 1.04)	1.05 (0.99, 1.11)	0.97 (0.92, 1.01)
Physical Activity Self—Efficacy Mean (continuous, per unit)	1.13 (0.93, 1.36)	1.61 (0.91, 2.84)	1.12 (0.80, 1.55)
_cons	0.27 (0.05, 1.37)	0.01 (0.00, 1.00)	0.42 (0.01, 12.32)
Pseudo r-squared	0.15	0.32	0.10

* Only participants with no missing data included in analyses.

**Table 4:** Multiple linear regression of socio-demographic, health, environmental and psychological associations of those that engage in `some` active travel (continuous data) for full cohort and by low and high income.

Variable	Full Cohort (*n* = 609) *	Low Income (*n* = 126) *	High Income (*n* = 202) *
	Coef. (95% CI)	Coef. (95% CI)	Coef. (95% CI)
Sex			
Male	31.77 (−10.99, 74.54)	27.92 (−86.96, 142.80)	28.42 (−39.70, 96.55)
Female	Ref.	Ref.	Ref.
Age (continuous, per year)	0.58 (-1.16, 2.32)	0.38 (-3.19, 3.95)	1.87 (-1.37, 5.11)
Marital status			
Married/co-habiting	20.36 (−27.80, 68.53)	25.20 (−94.33, 144.72)	80.04 (0.16, 159.92)
Single/Separated/divorced/widowed	Ref.	Ref.	Ref.
Housing tenure			
Owned outright	−48.63 (−115.40, 18.13)	−10.02 (−162.03, 142.00)	−177.86 (−302.11, −53.61)
Mortgage/co-ownership	−21.38 (−82.43, 39.67)	97.13 (−133.36, 327.62)	−122.72 (−224.50, −20.95)
Rented/other	Ref.	Ref.	Ref.
Weekly household income			
Low Income (£60–£230)	2.15 (−73.93, 78.23)	-	-
Medium Income (£231–£580)	37.17 (−14.30, 88.64)	-	-
High Income (£581+)	Ref.	-	-
BMI category			
Underweight/Normal	5.02 (−36.63, 46.67)	−5.22 (−114.26, 103.82)	22.21 (−44.57, 88.99)
Overweight/Obese	Ref.	Ref.	Ref.
Highest education			
Primary—none/other qualifications	16.86 (−58.12, 91.83)	−22.38 (−207.53, 162.77)	−111.69 (−300.14, 76.76)
Secondary—GCSE/A-level	2.11 (−44.47, 48.68)	12.05 (−147.13, 171.24)	−39.71 (−117.92, 38.49)
Tertiary—degree/higher education	Ref.	Ref.	Ref.
Access to bicycle			
Yes	−4.71 (−51.22, 41.81)	31.15 (−124.81, 187.12)	−52.39 (−119.43, 14.64)
No	Ref.	Ref.	Ref.
Number of cars/vans owned			
0	50.54 (−24.73, 125.80)	−71.59 (−355.29, 212.11)	−52.47 (−237.51, 132.58)
1	−9.15 (−63.14, 44.85)	−202.40 (−470.54, 65.75)	−28.32 (−101.71, 45.08)
2+	Ref.	Ref.	Ref.
Employment			
Unemployed/economically inactive	73.60 (−41.19, 188.38)	130.67 (−206.70, 468.04)	136.23 (−17.57, 290.03)
Employed	Ref.	Ref.	Ref.
Long term illness limiting activities			
Yes	45.37 (−22.72, 113.46)	124.62 (−29.32, 278.56)	70.40 (−41.91, 182.71)
No	Ref.	Ref.	Ref.
Difficulty walking a quarter mile			
Yes	−59.48 (−136.21, 17.25)	−77.48 (−223.44, 68.48)	−67.39 (−207.98, 73.19)
No	Ref.	Ref.	Ref.
Distance travelled to work daily			
Do not work or study/Work at home	−63.65 (−176.99, 49.69)	−153.61 (−480.33, 173.11)	−73.94 (−225.90, 78.02)
Travel any distance to work	Ref.	Ref.	Ref.
Stage of change			
Pre-contemplation	−96.04 (−151.46, −40.61)	−55.07 (−194.67, 84.53)	−70.41 (−157.84, 17.03)
Contemplation/Preparation	−89.58 (−142.71, −18.46)	−224.41 (−381.06, −67.75)	−72.57 (−161.87, 16.73)
Action/Maintenance	Ref.	Ref.	Ref.
WEMWBS (continuous, per unit)	−1.55 (−4.59, 1.50)	−3.20 (−10.36, 3.95)	−3.86 (−9.23, 1.52)
SF8 Mental Summary Score (continuous, per unit)	1.96 (−1.07, 5.00)	2.84 (−4.67, 10.36)	3.95 (−1.64, 9.53)
SF8 Physical Summary Score (continuous, per unit)	3.30 (0.30, 6.30)	5.58 (−3.04, 14.19)	3.79 (−0.63, 8.21)
Physical Activity Self—Efficacy Mean (continuous, per unit)	23.11 (−1.69, 47.91)	88.85 (20.49, 157.21)	26.00 (−10.91, 62.91)
_cons	−11.22 (−229.84, 207.41)	−79.51 (−698.47, 539.46)	−0.59 (−358.09, 356.91)
R-squared	0.11	0.28	0.18

* Only participants with no missing data were included in analyses.

**Table 5:** Multiple logistic regression of socio-demographic, health, environmental and psychological associations of those engaging in 150 min plus of active travel for full cohort and by low and high income.

Variable	Full Cohort (*n* = 916) *	Low Income (*n* = 219) *	High Income (*n* = 277) *
	Odds Ratio (95% CI)	Odds Ratio (95% CI)	Odds Ratio (95% CI)
Sex			
Male	1.11 (0.82, 1.52)	1.42 (0.63, 3.18)	1.53 (0.87, 2.72)
Female	Ref.	Ref.	Ref.
Age (continuous, per year)	0.98 (0.97, 0.99)	0.98 (0.96, 1.01)	0.99 (0.96, 1.02)
Marital status			
Married/co-habiting	1.42 (0.98, 2.05)	6.50 (2.15, 19.65)	1.49 (0.73, 3.06)
Single/Separated/divorced/widowed	Ref.	Ref.	Ref.
Housing tenure			
Owned outright	1.27 (0.77, 2.07)	1.79 (0.59, 5.44)	0.51 (0.17, 1.54)
Mortgage/co-ownership	1.02 (0.65, 1.60)	7.84 (1.22, 50.24)	0.45 (0.18, 1.11)
Rented/other	Ref.	Ref.	Ref.
Weekly household income			
Low Income (£60–£230)	1.08 (0.60, 1.95)	-	-
Medium Income (£231–£580)	1.33 (0.90, 1.97)	-	-
High Income (£581+)	Ref.	-	-
BMI category			
Underweight/Normal	0.89 (0.66, 1.22)	0.45 (0.20, 1.04)	1.80 (1.02, 3.18)
Overweight/Obese	Ref.	Ref.	Ref.
Highest education			
Primary—none/other qualifications	0.79 (0.46, 1.35)	2.34 (0.59, 9.31)	1.03 (0.19, 5.50)
Secondary—GCSE/A-level	0.87 (0.62, 1.23)	3.41 (0.97, 12.04)	0.47 (0.24, 0.91)
Tertiary—degree/higher education	Ref.	Ref.	Ref.
Access to bicycle			
Yes	1.06 (0.75, 1.51)	0.68 (0.21, 2.19)	1.13 (0.64, 1.98)
No	Ref.	Ref.	Ref.
Number of cars/vans owned			
0	2.26 (1.27, 4.03)	4.27 (0.47, 39.07)	1.62 (0.31, 8.38)
1	1.35 (0.90, 2.03)	1.19 (0.15, 9.43)	1.27 (0.68, 2.36)
2+	Ref.	Ref.	Ref.
Employment			
Unemployed/economically inactive	2.32 (1.00, 5.40)	1.35 (0.16, 11.02)	3.11 (0.82, 11.75)
Employed	Ref.	Ref.	Ref.
Long term illness limiting activities			
Yes	1.58 (0.95, 2.65)	3.64 (1.08, 12.24)	0.90 (0.34, 2.36)
No	Ref.	Ref.	Ref.
Difficulty walking a quarter mile			
Yes	0.41 (0.23, 0.74)	0.25 (0.08, 0.76)	0.95 (0.28, 3.28)
No	Ref.	Ref.	Ref.
Distance travelled to work daily			
Do not work or study/Work at home	0.66 (0.29, 1.51)	0.45 (0.06, 3.55)	0.67 (0.18, 2.41)
Travel any distance to work	Ref.	Ref.	Ref.
Stage of change			
Pre-contemplation	0.36 (0.23, 0.55)	0.23 (0.08, 0.66)	0.73 (0.35, 1.55)
Contemplation/Preparation	0.28 (0.18, 0.44)	0.06 (0.01, 0.24)	0.40 (0.19, 0.84)
Action/Maintenance	Ref.	Ref.	Ref.
WEMWBS (continuous, per unit)	1.02 (1.00, 1.05)	1.03 (0.98, 1.08)	1.02 (0.97, 1.07)
SF8 Mental Summary Score (continuous, per unit)	1.01 (0.99, 1.03)	1.01 (0.95, 1.06)	1.03 (0.98, 1.07)
SF8 Physical Summary Score (continuous, per unit)	1.03 (1.01, 1.06)	1.06 (0.99, 1.13)	1.01 (0.97, 1.06)
Physical Activity Self—Efficacy Mean (continuous, per unit)	1.02 (0.85, 1.22)	1.20 (0.72, 1.99)	1.05 (0.77, 1.42)
_cons	0.06 (0.01, 0.34)	0.00 (0.00, 0.41)	0.05 (0.00, 1.18)
Pseudo R-squared	0.16	0.34	0.11

* Only participants with no missing data included in analyses.

## Data Availability

The data presented in this study are available on reasonable request from the corresponding author.

## Supplementary Materials

The following are available online at www.mdpi.com/1660-4601/181/91/360/s1, Table S1. Table of participant socio-demographics for medium income; Table S2; Engagement in active travel by participants for medium income; Table S3; Multiple logistic regression of socio-demographic, health, environmental and psychological associations of none/some active travel (binary) for medium income; Table S4; Multiple linear regression of socio-demographic, health, environmental and psychological associations of those that engage in `some` active travel (continuous data) for medium income (i.e., those that said they do 10 min or more); Table S5; Multiple logistic regression of socio-demographic, health, environmental and psychological associations of those engaging in 150+ min for medium income; Table S6; Multiple imputation chain equation of socio-demographic, health, environmental and psychological associations of none/some active travel (binary) for full cohort Table S7; Multiple imputation chain equation of socio-demographic, health, environmental and psychological associations of those that engage in `some` active travel (continuous data) for full cohort; Table S8; Multiple imputation chain equation of socio-demographic, health, environmental and psychological associations of those engaging in 150 minutes plus of active travel for full cohort.
