# Supplementary material for: Individual Characteristics Associated with Active Travel in Low and High Income Groups in the UK
Source: Int J Environ Res Public Health. 2021 Oct 1;18(19):10360. doi: 10.3390/ijerph181910360 (PMC8508371; doi:10.3390/ijerph181910360)
Supplement: Supplementary file 1 [file ijerph-18-10360-s001.zip › ijerph-1298672-supplementary.pdf]

Table S1. Table of participant socio-demographics for medium income

Variable	N (Total n=441)	%
**Sex**		
Male	218	49.43
Female	223	50.57
**Age**		
16 to 39 years	159	36.05
40 to 64 years	155	35.15
65+ years	127	28.80
Mean (years):	51.30 (SD 18.85)	
Range (years):	16-99	
**Marital status***		
Married/co-habiting	206	46.71
Single/ Separated/divorced/widowed	235	53.29
**Housing tenure**		
Owned outright	159	36.05
Mortgage/ co-ownership	93	21.09
Rented/other	189	42.86
**BMI category ***		
<24.9 Underweight / Normal	186	43.56
25 – 29.9 Overweight /> 30 Obese	241	56.44
**Highest education**		
Primary- none/ other qualifications	64	14.51
Secondary – GCSE/ A-level	188	42.63
Tertiary – degree/ higher education	189	42.86
**Access to bicycle**		
Yes	118	26.76
No	323	73.24
**Number of cars/vans owned**		
0	112	25.40
1	236	53.51
2+	93	21.09
**Employment**		
Employed	249	56.46
Unemployed/ economically inactive	192	43.54
**Long term illness limiting activities**		
Yes	110	24.94
No	331	75.06
**Difficulty walking a quarter mile**		
Yes	77	17.46
No	364	82.54
**Distance travelled to work daily**		
Do not work or study/ Work at home	198	44.90
Travel any distance to work	243	55.10
**Active travel**		
None	149	33.79
Some	292	66.21
**Stage of change**		
Pre-contemplation	138	31.29
Contemplation/ Preparation	50	11.34
Action/ Maintenance	253	57.37
	Mean	*SD*
**WEMWBS**	52.54	8.75
**SF8 Mental Summary Score**	49.97	10.16
**SF8 Physical Summary Score**	49.17	10.94
**Physical Activity Self – Efficacy Mean ***	2.35	0.90

Table S2; Engagement in active travel by participants for medium income

**Variable**	
Median minutes of active travel per week (minutes (IQR))	120 (0-280)
Number of participants doing `some` active travel (over 10 minutes/per week) (n (%))	292 (66.21)
Median minutes of active travel for those that do `some` active travel' (over 10 minutes/per week) (minutes (IQR))	210 (120-360)
Number of participants that achieve over 150 minutes of active travel per week (n (%))	205 (46.49)

IQR: Inter-quartile range

Table S3; Multiple logistic regression of socio-demographic, health, environmental and psychological associations of none/some active travel (binary) for medium income

Variable	Odds ratio	95% CI
**Sex**		
Male	0.80	0.49, 1.31
Female	Ref.	Ref.
**Age** (continuous)	0.98	0.96, 1.00
**Marital status**		
Married/co-habiting	1.05	0.62, 1.79
Single/ Separated/ divorced/widowed	Ref.	Ref.
**Housing tenure**		
Owned outright	2.18	1.02, 4.67
Mortgage/ co-ownership	1.87	0.92, 3.82
Rented/other	Ref.	Ref
**Weekly household income**		
Low Income (£60 - £230)	-	-
Medium Income (£231 - £580)	-	-
High Income (£581+)	-	-
**BMI category**		
Underweight/ Normal	0.63	0.39, 1.03
Overweight/Obese	Ref.	Ref.
**Highest education**		
Primary- none/ other qualifications	0.60	0.28, 1.28
Secondary – GCSE/ A-level	0.90	0.53, 1.51
Tertiary – degree/ higher education	Ref.	Ref.
**Access to bicycle**		
Yes	1.18	0.65, 2.16
No	Ref.	Ref.
**Number of cars/vans owned**		
0	1.61	0.68, 3.82
1	0.80	0.41, 1.56
2+	Ref.	Ref.
**Employment**		
Unemployed/ economically inactive	1.41	0.33, 6.10
Employed	Ref.	Ref.
**Long term illness limiting activities**		
Yes	0.91	0.41, 2.06
No	Ref.	Ref.
**Difficulty walking a quarter mile**		
Yes	0.31	0.12, 0.76
No	Ref.	Ref.
**Distance travelled to work daily**		
Do not work or study/ Work at home	0.80	0.19, 3.37
Travel any distance to work	Ref.	Ref.
**Stage of change**		
Pre-contemplation	0.54	0.28, 1.03
Contemplation/ Preparation	0.41	0.20, 0.85
Action/ Maintenance	Ref.	Ref.
**WEMWBS**	1.03	0.99, 1.06
**SF8 Mental Summary Score**	0.99	0.96, 1.02
**SF8 Physical Summary Score**	1.01	0.98, 1.05
**Physical Activity Self – Efficacy Mean**	0.91	0.68, 1.23
**_cons**	2.84	0.22, 36.94
**Pseudo r-squared**	0.17	

*Only participants with no missing data included in analyses

Table S4; Multiple linear regression of socio-demographic, health, environmental and psychological associations of those that engage in `some` active travel (continuous data) for medium income

Variable	Coef.	95% CI
**Sex**		
Male	37.94	-31.50, 107.37
Female	Ref.	Ref.
**Age** (continuous)	0.73	-2.09, 3.54
**Marital status**		
Married/co-habiting	-11.66	-84.91, 61.60
Single/ Separated/ divorced/widowed	Ref.	Ref.
**Housing tenure**		
Owned outright	-6.88	-111.66, 97.91
Mortgage/ co-ownership	25.63	-64.52, 115.77
Rented/other	Ref.	Ref.
**Weekly household income**		
Low Income (£60 - £230)	-	-
Medium Income (£231 - £580)	-	-
High Income (£581+)	-	-
**BMI category**		
Underweight/ Normal	-20.14	-85.52, 45.23
Overweight/Obese	Ref.	Ref.
**Highest education**		
Primary- none/ other qualifications	157.36	33.65, 281.06
Secondary – GCSE/ A-level	12.48	-54.88, 79.83
Tertiary – degree/ higher education	Ref.	Ref.
**Access to bicycle**		
Yes	14.31	-59.84, 88.67
No	Ref.	Ref.
**Number of cars/vans owned**		
0	95.45	-15.71, 206.61
1	34.30	-52.39, 121.00
2+	Ref.	Ref.
**Employment**		
Unemployed/ economically inactive	15.93	-187.70, 219.57
Employed	Ref.	Ref.
**Long term illness limiting activities**		
Yes	26.49	-89.15, 142.13
No	Ref.	Ref.
**Difficulty walking a quarter mile**		
Yes	-49.20	-192.92, 94.53
No	Ref.	Ref.
**Distance travelled to work daily**		
Do not work or study/ Work at home	-33.07	-239.97, 173.83
Travel any distance to work	Ref.	Ref.
**Stage of change**		
Pre-contemplation	-116.81	-204.66, -28.96
Contemplation/ Preparation	-46.05	-154.62, 62.52
Action/ Maintenance	Ref.	Ref.
**WEMWBS**	2.26	-2.35, 6.87
**SF8 Mental Summary Score**	-0.99	-5.50, 3.53
**SF8 Physical Summary Score**	3.47	-1.68, 8.62
**Physical Activity Self – Efficacy Mean**	7.30	-33.67, 48.26
**_cons**	-51.86	-405.98, 302.26
**R-squared**	0.11	

*Only participants with no missing data included in analyses

Table S5; Multiple logistic regression of socio-demographic, health, environmental and psychological associations of those engaging in 150+ minutes of active travel for medium income

Variable	Odds ratio	95% CI
**Sex**		
Male	0.90	0.56, 1.44
Female	Ref.	Ref.
**Age** (continuous)	0.97	0.95, 0.99
**Marital status**		
Married/co-habiting	1.15	0.69, 1.92
Single/ Separated/ divorced/widowed	Ref.	Ref.
**Housing tenure**		
Owned outright	1.78	0.84, 3.77
Mortgage/ co-ownership	1.22	0.64, 2.33
Rented/other	Ref.	Ref.
**Weekly household income**	-	-
Low Income (£60 - £230)	-	-
Medium Income (£231 - £580)	-	-
High Income (£581+)		
**BMI category**		
Underweight/ Normal	0.69	0.44, 1.11
Overweight/Obese	Ref.	Ref.
**Highest education**		
Primary- none/ other qualifications	0.89	0.40, 2.00
Secondary – GCSE/ A-level	0.96	0.59, 1.55
Tertiary – degree/ higher education	Ref.	Ref.
**Access to bicycle**		
Yes	1.05	0.62, 1.81
No	Ref.	Ref.
**Number of cars/vans owned**		
0	2.34	1.05, 5.21
1	1.17	0.63, 2.16
2+	Ref.	Ref.
**Employment**		
Unemployed/ economically inactive	2.66	0.60, 11.82
Employed	Ref.	Ref.
**Long term illness limiting activities**		
Yes	1.70	0.73, 3.98
No	Ref.	Ref.
**Difficulty walking a quarter mile**		
Yes	0.24	0.09, 0.69
No	Ref.	Ref.
**Distance travelled to work daily**		
Do not work or study/ Work at home	0.65	0.15, 2.76
Travel any distance to work	Ref.	Ref.
**Stage of change**		
Pre-contemplation	0.26	0.14, 0.49
Contemplation/ Preparation	0.27	0.13, 0.56
Action/ Maintenance	Ref.	Ref.
**WEMWBS**	1.02	0.99, 1.06
**SF8 Mental Summary Score**	0.99	0.96, 1.02
**SF8 Physical Summary Score**	1.03	0.99, 1.07
**Physical Activity Self – Efficacy Mean**	0.91	0.68, 1.21
**_cons**	0.65	0.05, 8.60
**Pseudo R-squared**	0.19	

*Only participants with no missing data included in analyses

Table S6; Multiple imputation chain equation of socio-demographic, health, environmental and psychological associations of none/some active travel (binary) for full cohort

Variable	Coef.	95% CI
**Sex**		
Male	0.02	-0.26, 0.30
Female	Ref.	Ref.
**Age** (continuous)	**-0.02**	**-0.03, -0.01**
**Marital status**		
Married/co-habiting	0.06	-0.27, 0.40
Single/ Separated/ divorced/widowed	Ref.	Ref.
**Housing tenure**		
Owned outright	0.31	-0.10, 0.73
Mortgage/ co-ownership	0.30	-0.11, 0.72
Rented/other	Ref.	Ref.
**Weekly household income**		
Low Income (£60 - £230)	0.37	-0.23, 0.96
Medium Income (£231 - £580)	0.10	-0.31, 0.52
High Income (£581+)	Ref.	Ref.
**BMI category**		
Underweight/ Normal	**-0.39**	**-0.68, -0.11**
Overweight/Obese	Ref.	Ref.
**Highest education**		
Primary- none/ other qualifications	-0.42	-0.87, 0.03
Secondary – GCSE/ A-level	-0.31	-0.64, 0.02
Tertiary – degree/ higher education	Ref.	Ref.
**Access to bicycle**		
Yes	0.15	-0.21, 0.50
No	Ref.	Ref.
**Number of cars/vans owned**		
0	0.52	0.00, 1.04
1	0.31	-0.07, 0.69
2+	Ref.	Ref.
**Employment**		
Unemployed/ economically inactive	-0.11	-0.75, 0.52
Employed	Ref.	Ref.
**Long term illness limiting activities**		
Yes	0.21	-0.23, 0.65
No	Ref.	Ref.
**Difficulty walking a quarter mile**		
Yes	**-1.00**	**-1.45, -0.54**
No	Ref.	Ref.
**Distance travelled to work daily**		
Do not work or study/ Work at home	0.26	-0.40, 0.93
Travel any distance to work	Ref.	Ref.
**Stage of change**		
Pre-contemplation	**-0.72**	**-1.10, -0.34**
Contemplation/ Preparation	**-0.79**	**-1.16, -0.41**
Action/ Maintenance	Ref.	Ref.
**WEMWBS**	**0.03**	**0.01, 0.05**
**SF8 Mental Summary Score**	0.00	-0.01, 0.02
**SF8 Physical Summary Score**	0.01	-0.00, 0.03
**Physical Activity Self – Efficacy Mean**	0.13	-0.04, 0.30
**_cons**	-0.95	-2.36, 0.46

Table S7; Multiple imputation chain equation of socio-demographic, health, environmental and psychological associations of those that engage in `some` active travel (continuous data) for full cohort

Variable	Coef.	95% CI
**Sex**		
Male	25.17	-15.25, 65.58
Female	Ref.	Ref.
**Age** (continuous)	0.38	-1.25, 2.00
**Marital status**		
Married/co-habiting	19.08	-26.92, 65.08
Single/ Separated/ divorced/widowed	Ref.	Ref.
**Housing tenure**		
Owned outright	-53.76	-116.56, 9.04
Mortgage/ co-ownership	-24.37	-80.97, 32.23
Rented/other	Ref.	Ref.
**Weekly household income**		
Low Income (£60 - £230)	22.59	-62.79, 107.96
Medium Income (£231 - £580)	29.17	-27.67, 86.01
High Income (£581+)	Ref.	Ref.
**BMI category**		
Underweight/ Normal	14.60	-25.74, 54.95
Overweight/Obese	Ref.	Ref.
**Highest education**		
Primary- none/ other qualifications	-2.91	-72.87, 67.05
Secondary – GCSE/ A-level	-10.37	-54.90, 34.16
Tertiary – degree/ higher education	Ref.	Ref.
**Access to bicycle**		
Yes	-25.13	-69.60, 19.34
No	Ref.	Ref.
**Number of cars/vans owned**		
0	45.05	-25.44, 115.55
1	-12.53	-63.34, 38.27
2+	Ref.	Ref.
**Employment**		
Unemployed/ economically inactive	21.54	-57.55, 100.62
Employed	Ref.	Ref.
**Long term illness limiting activities**		
Yes	17.99	-45.28, 81.27
No	Ref.	Ref.
**Difficulty walking a quarter mile**		
Yes	-44.99	-119.82, 29.84
No	Ref.	Ref.
**Distance travelled to work daily**		
Do not work or study/ Work at home	-31.17	-115.13, 52.78
Travel any distance to work	Ref.	Ref.
**Stage of change**		
Pre-contemplation	**-92.69**	**-146.56, -38.81**
Contemplation/ Preparation	**-107.36**	**-163.89, -50.83**
Action/ Maintenance	Ref.	Ref.
**WEMWBS**	-0.66	-3.54, 2.22
**SF8 Mental Summary Score**	1.14	-1.72, 4.00
**SF8 Physical Summary Score**	**3.13**	**0.25, 6.00**
**Physical Activity Self – Efficacy Mean**	**25.98**	**2.89, 49.08**
**_cons**	26.98	-181.79, 235.75

Table S8; Multiple imputation chain equation of socio-demographic, health, environmental and psychological associations of those engaging in 150 minutes plus of active travel for full cohort

Variable	Coef.	95% CI
**Sex**		
Male	0.10	-0.18, 0.37
Female	Ref.	Ref.
**Age** (continuous)	-0.01	-0.03, -0.00
**Marital status**		
Married/co-habiting	**0.36**	**0.04, 0.69**
Single/ Separated/ divorced/widowed	Ref.	Ref.
**Housing tenure**		
Owned outright	0.14	-0.29, 0.57
Mortgage/ co-ownership	0.05	-0.34, 0.44
Rented/other	Ref.	Ref.
**Weekly household income**		
Low Income (£60 - £230)	0.14	-0.41, 0.69
Medium Income (£231 - £580)	0.21	-0.18, 0.61
High Income (£581+)	Ref.	Ref.
**BMI category**		
Underweight/ Normal	-0.16	-0.43, 0.12
Overweight/Obese	Ref.	Ref.
**Highest education**		
Primary- none/ other qualifications	-0.30	-0.77, 0.17
Secondary – GCSE/ A-level	-0.20	-0.51, 0.11
Tertiary – degree/ higher education	Ref.	Ref.
**Access to bicycle**		
Yes	0.07	-0.25, 0.38
No	Ref.	Ref.
**Number of cars/vans owned**		
0	**0.78**	**0.28, 1.28**
1	0.24	-0.12, 0.60
2+	Ref.	Ref.
**Employment**		
Unemployed/ economically inactive	0.23	-0.34, 0.79
Employed	Ref.	Ref.
**Long term illness limiting activities**		
Yes	0.41	-0.03, 0.85
No	Ref.	Ref.
**Difficulty walking a quarter mile**		
Yes	**-1.10**	**-1.63, -0.58**
No	Ref.	Ref.
**Distance travelled to work daily**		
Do not work or study/ Work at home	0.01	-0.58, 0.61
Travel any distance to work	Ref.	Ref.
**Stage of change**		
Pre-contemplation	**-1.08**	**-1.47, -0.70**
Contemplation/ Preparation	**-1.41**	**-1.80, -1.01**
Action/ Maintenance	Ref.	Ref.
**WEMWBS**	0.02	-0.00, 0.04
**SF8 Mental Summary Score**	0.00	-0.01, 0.02
**SF8 Physical Summary Score**	**0.03**	**0.01, 0.05**
**Physical Activity Self – Efficacy Mean**	0.02	-0.14, 0.18
**_cons**	-2.33	-3.78, -0.87
